# Supplementary material for: Cardiodynamic variables measured by impedance cardiography during a 6-minute walk test are reliable predictors of peak oxygen consumption in young healthy adults
Source: PLoS One. 2021 May 25;16(5):e0252219. doi: 10.1371/journal.pone.0252219 (PMC8148309; doi:10.1371/journal.pone.0252219)
Supplement: S1 Table — Data are mean±SD. 6MWT = 6 minute walk test; CPET = cardiopulmonary exercise test; SpO2 = oxygen saturation measured by oximeter; SBP = systolic blood pressure; DBP = diastolic blood pressure. (DOCX) [file pone.0252219.s001.docx]

S1 Table. Oxygen saturation and blood pressures at baseline, immediately post and 10 min post-CPET and post-6MWT. Data are mean±SD

|  | 6MWT | | | CPET | | |
| --- | --- | --- | --- | --- | --- | --- |
|  | Baseline | Immediately post-test | 10-min  Post-test | Baseline | Immediately post-test | 10-min  Post-test |
| SpO_2_ (%) | 98±0 | 98±1 | 98±1 | 98±0 | 97±1 | 98±1 |
| SBP (mmHg) | 110±10 | 127±15 | 108±13 | 108±7 | 163±18 | 106±9 |
| DBP  (mmHg) | 69±9 | 77±11 | 67±11 | 67±9 | 70±11 | 68±12 |

6MWT = 6 minute walk test; CPET = cardiopulmonary exercise test; SpO2 = oxygen saturation measured by oximeter; SBP=systolic blood pressure; DBP=diastolic blood pressure
