# Supplementary material for: Cardiodynamic variables measured by impedance cardiography during a 6-minute walk test are reliable predictors of peak oxygen consumption in young healthy adults
Source: PLoS One. 2021 May 25;16(5):e0252219. doi: 10.1371/journal.pone.0252219 (PMC8148309; doi:10.1371/journal.pone.0252219)
Supplement: Attachment — Submitted filename: Responses to reviewers comments.docx [file pone.0252219.s002.docx]

**Responses to reviewers**

*We are grateful for all our reviewers’ valuable and insightful comments and have modified our manuscript following their suggestions.*

**Reviewer #1:**

The manuscript "Cardiodynamic variables measured by Impedance Cardiography during a 6-minute walk test are reliable predictors of peak oxygen consumption in young healthy adults" is very interesting and of high clinical utility. The design of the s

tudy is correct and thoughtful, the aim is clear, the results support the conclusions. The discussion is consistent and balanced. The proposed 6MWT/ICG and elaborated equation for calculation of VO2peak could be used in practice, i.e. for screening exams in young subjects. At first glance simply study effected in really valuable conclusions.

I have only minor comments to be considered:
1. Introduction, verse 38 "....measured data during a 6MWT will be a stronger predictor of...." - maybe better "....measured data during a 6MWT will produce a stronger predictor of...."
2. Methods, verse 68-70 - the sentence about MasterScreen CPX - please delete here and insert in sentence verse 85-86 in CPET description.

*Authors responses:*

*We are grateful for Reviewer 1’s supportive comments. We have replaced ‘be’ with ‘produce’ in the sentence as advised. Please see line 43 under Introduction.*

*We have now moved the description of MasterScreen CPX to the section under CPET, please see line 93.*

**Reviewer #2:**

The present study investigates the use of impedance cardiography during a 6MWT for better predictability of VO2max. In general, the study seems well conducted and the manuscript is clear and easy to read. The authors should be complemented for addressing an important issue in the assessment of physical fitness in the clinical setting. I have only few comments for consideration.

*Authors response: We are grateful for Reviewer 2’s encouraging comments*

Abstract:
- Line 10-21: A presentation of the peak values HR, SV, CO in CEPT and 6MWT would contribute to a better understanding of the study in the abstract. The description of the regression analyses could be more concise.

*Authors response: The peak values of HR, SV, CO in CPET and 6MWT are now included in the Abstract. Units in the equation were removed so the equation now appears more concise.*

Introduction:
- Lines 32-34: A little more detail is needed regarding the prediction of oxygen consumption due to the 6MWT.

*Authors response: An extra sentence is now included in the Introduction to highlight the relationship between oxygen consumption and ICG cardiodynamic data during 6MWT. Please see lines 36-39.*

Methods:
- Line 73: Why were two consecutive 6MWT conducted?

*Authors response: the reason for conducting two 6MWTs is now included, please refer to lines 78-81.*

Discussion:
- Line 318: Measuring VO2 with a portable spirometry during the 6MWT would also be useful.

*Authors response: This is an excellent point. We were aware of the advantage of using a portable telemetric device to measure oxygen consumption during 6MWT. Unfortunately, we did not have the resources to acquire this equipment. This has now been addressed as a limitation. Please see lines 328-331.*

Also, the feasibility nature of the study should be addressed. The authors have demonstrated the benefits of impedance cardiography in simple clinical examinations. Further investigations (e.g. before and after operations) in various patient cohorts would be useful in the sequel. I recommend considering the manuscript for publication.

*Authors response: we have now included the feasibility point in the section “Implications of our study’, please refer to lines 319-321.*

**Reviewer #3:**

In the manuscript "Cardiodynamic variables measured by Impedance Cardiography during a 6-minute walk test are reliable predictors of peak oxygen consumption in young healthy adults" authors determine whether prediction of VO2peak can be improved by the inclusion of cardiovascular indices derived by impedance cardiography (ICG) during the 6MWT.
This manuscript is correctly written, with the connection between aim, results, discussion and conclusions preserved. In my opinion, it is of significant clinical importance, although it is very limited to the studied age group, without chronic diseases, according to the authors' opinion.

*Authors response: We are thankful for Reviewer 3’s positive comments. We are currently extending our study to include subjects after stroke.*

Minor comments:
1. While planning training or rehabilitation, it is important to determine the anaerobic threshold. Do you have any data on the level of effort achieved during the 6MWT (line 273-submaximal)? At the end of 6MWT, does the patient get VO2 corresponding to peak VO2 or VO2 at anaerobic threshold (AT) during CPET? Hence, there may be a difference in the strength of the correlation between hemodynamic parameters and VO2 between CPET and 6MWT. The incresases in SV and HR from their values at rest to those at the AT (AT–rest) are similar, but the increase in SV is significantly lower than HR after AT. Moreover, the increase in SV after AT is sensitive to lack of training and chronic diseases. Please complete the discussion on line 260-269.

*Authors response: Thank you for this excellent comment. Indeed we have collected data on VO2 at anaerobic threshold during CPET. We have reported that the RPE level achieved at the end of CPET was 8/10 and that for 6MWT was 7/10 (line 250) but we did not report our AT data because the correlation between cardiodynamic and VO2peak data was much stronger than correlation with VO2 at AT. As suggested by the reviewer, we now consider that this information would be a nice addition to our report. We have therefore included data recorded at AT in Tables 3 and 4, and in the Results section: lines 182-184, and the Discussion at lines 263-269 and lines 276-277.*

2. How did you choose values for hemodynamic parameters suitable for analysis? How did you deal with measurement errors? Please complete the methodology - line 103.

*Authors response: Thank you for pointing this out. We averaged the final 10 seconds of the ICG data recorded to provide the peak cardiodynamic variables of the 6MWT, and data for CPET were computed similarly. This measurement method was considered reliable as previously reported [9]. We have now included this in the Methodology section, lines 112-114.*
